# Plasma screening in mid-charged ions observed by K-shell line emission

**DOI:** 10.1038/s41598-026-39041-1

**Published:** 2026-02-10

**Authors:** M. Šmíd, O. S. Humphries, C. Baehtz, V. Bouffetier, E. Brambrink, T. Burian, V. Cerantola, M. S. Cho, T. E. Cowan, L. Gaus, M. F. Gu, V. Hájková, L. Juha, J. Kaa, Z. Konopkova, M. Kozlová, H. P. Le, M. Makita, X. Pan, T. R. Preston, A. Schropp, J.-P. Schwinkendorf, H. A. Scott, R. Štefaníková, J. Vorberger, W. Wang, U. Zastrau, K. Falk

**Affiliations:** 1 Helmholtz Zentrum Dresden Rossendorf, Bautzner Landstraße 400, 01328 Dresden, Germany; 2 European XFEL, Holzkoppel 4, 22869 Schenefeld, Germany; 3 Institute of Physics, Czech Academy of Sciences, Na Slovance 2, 182 00 Praha, Czech Republic; 4 Department of Earth and Environmental Sciences, University of Milano-Bicocca, Piazza della Scienza 4, 20126 Milano, Italy; 5 Lawrence Livermore National Laboratory, 7000 East Avenue, Livermore, CA 94550 USA; 6 Space Science Laboratory, University of California, Berkeley, CA 94720 USA; 7 Institute of Plasma Physics of the Czech Academy of Sciences, U Slovanky 2525/1a, 182 00 Praha, Czech Republic; 8 Technische Universität Dresden, 01062 Dresden, Germany; 9 Centre for X-ray and Nano Science CXNS, Deutsches Elektronen-Synchrotron DESY, Notkestrasse 85, 22607 Hamburg, Germany

**Keywords:** Materials science, Physics

## Abstract

Dense plasma environment affects the electronic structure of ions via variations of the microscopic electrical fields, also known as *plasma screening*. This effect can be either estimated by simplified analytical models, or by computationally expensive and to date unverified numerical calculations. We have experimentally quantified plasma screening from the energy shifts of the bound-bound transitions in matter driven by the x-ray free electron laser (XFEL). This was enabled by identification of detailed electronic configurations of the observed K$\upalpha$, K$\upbeta$ and K$\upgamma$ lines. This work paves the way for improving plasma screening models including connected effects like ionization potential depression and continuum lowering, which will advance the understanding of atomic physics in the Warm Dense Matter regime.

## Introduction

Electrons bound in atoms are held at specific levels—shells and subshells. The energy of these levels is determined by the electric potential of the ion, which is influenced by the presence of other electrons, whether bound within the atom or freely moving in its immediate vicinity. The simplest way to measure this influence is through radiative atomic transitions, i.e., processes in which a bound electron moves from one level to another, accompanied by the emission or absorption of an X-ray photon with a wavelength exactly corresponding to the energy difference of the levels. Such transitions, including the Cu K$\upalpha$ line whose behaviour is studied in this work, were observed and characterized already in 1909^1^. Its wavelength (energy) was first measured in 1913^2^ with a deviation better than 3% to modern benchmarks^3^. Those observations actually led to the discovery of electronic structure of ions. Even today, observing changes in the energies of these transitions remains an excellent method for revealing the structure of atoms and their sensitivity to the surrounding environment. Among other effects, we can speak of line shifts due to two factors: the influence of bound electrons (electron configuration) and the influence of free electrons.

The understanding and quantitative analysis of both shifts relies on complex modelling, which depends on several approximations. One of those is the *plasma screening*, describing how the free electron environment affects the potential of the emitting ion. Most used models of plasma screening are based on the calculations from about 60 years ago^4,5^. The advent of X-ray free electron lasers (XFELs) opened up new possibilities to experimentally challenge those models, to observe plasma screening via a shift of the emission lines or absorption edges as a function of plasma conditions. Still, it is typically not straightforward to extract the *continuous* effect of screening, since the line shifts are at the same time influenced by the *discrete* changes of the electronic configuration, which is, similarly as screening, affected by plasma temperature.

The change of line position due to bound electrons, or, in other words, electronic configuration, can be well illustrated on the $1s-2p$ transition. Its energy is mostly influenced by the K-shell occupation, electrons in the L-shell have a smaller effect, and the influence of M- and N- shell electrons can be often neglected: The addition of a K-shell electron actually changes the name of the transition: so $1s^1-2p^1$ is called Ly$_\upalpha$, while additional electron leads to $1s^2 - 1s2p$ which is the He$_\alpha$. Adding an electron to the L-shell can produce $1s^2 2p - 1s2p^2$, which could be called either Li-like satellite of He$_\alpha$, or Li-like K$\upalpha$. Transitions from ions with more electrons can then be called K$\upalpha$ satellites. Those were first calculated in 1969^6^ as a function of the L-shell occupation, and experimentally observed in 1975^7^. However, the exact description of many-electron systems like Cu is computationally challenging, as the number of possible configurations of available electrons (29 in copper) is vast, and the lines are mostly indistinguishable. Such modelling of non-LTE plasmas was attempted with super-configuration codes, but the results still show large deviations from observations^8^. For example, in the atomic model presented in this paper, the number of K shell transitions is about 120 million, out of which about 1.5 million represents $1s-2p$ lines. In experiment, typically only 9 emission lines defined by the occupation of the L-shell are resolvable. These *heated* K$\upalpha$ satellite lines have vast applications at Warm Dense Matter (WDM) and plasma diagnostics, as identified already in 1981^9^, their recent applications are shown e.g. in^10–14^. The advantage of those lines is that their emission is produced by highly charged ions present in temperatures of hundreds to thousands of eV, while their emission energies lie in a narrow range well resolvable by high-resolution crystal spectrometers, therefore providing a unique insight into the plasma conditions. The proper understanding of x-ray spectroscopy and atomic physics is also important for fusion research^15^.

The second effect altering the line position is the *plasma screening*^16^, occurring when the free electrons surrounding the ion alter its electric field. The screening has several consequences: The change of transition energy is often called *Stark shift*. The *ionization potential depression* (IPD) describes the decrease of energy needed to remove a bound electron into continuum, most often manifested in the form of shift of the absorption edge. *Continuum lowering* (CL)^17^ shows that the boundary between free electrons (continuum) and bound ones is decreasing, and therefore outer shells are effectively disappearing—merging into continuum. These effects are most often described by the Stewart-Pyatt model (SP)^5^ from 1966. Some recent experiments identifying the shifts of the K-edge indicated that the modified Ecker-Kröll model^4^ fits the measurements better^16,18,19^ or where the SP model fits closer^20^, spurring further model development^16,21,22^ and discussions^23^.

A pioneering experiment studying continuum lowering in atoms in dense plasma environment isochorically heated by XFEL beam at the LCLS laboratory have been shown in 2012^18^. The shift of absorption edges was measured in low-Z materials (Al, Mg, Si). The shift in Al was later calculated by using Density functional theory (DFT), with results in perfect agreement to the experimental data with charge states 3–7^24^. One recent approach to quantify IPD was shown in^21^, where the electron distribution was modelled by classical molecular dynamics. The averaged effect over an ensemble of configurations was then calculated, and shown to agree well with the previous experimental data up till charge state 9. However, the spectral simulations can completely skip the concept of IPD, as shown in^16^. Here, the DFT-based multi-band kinetic model (VERITAS) explicitly accounts for the interactions among ions and the dense plasma environment. Energy band shifting and ionization balance are therefore self-consistently calculated, without invocation of an ad hoc CL or IPD model. Such models are extremely computationally expensive and therefore it might be difficult to apply them to the complex atomic structures like those presented in this paper.

Recent first-principles studies have further clarified ionization potential depression in dense plasmas. A parameter-free quantum-mechanical treatment that successfully reproduces measured IPD in aluminum and resolves discrepancies among traditional continuum-lowering models was developed^25^. A generalized ion-sphere approach with spectroscopic accuracy was later introduced to calculate X-ray transition and K-edge energies in dense plasmas, enabling self-consistent predictions of edge shifts at finite temperature and density^26^. More recently, models incorporating bound–continuum mixing and band-structure effects have demonstrated improved agreement with experimental IPDs in low-Z materials, emphasizing the role of many-body effects in dense plasma spectroscopy^27^.

In 2012, Hu et al. pointed out that *“Detailed spectroscopic measurements at warm dense matter conditions are rare, and traditional collisional-radiative equilibrium models, based on isolated-atom calculations and ad hoc continuum lowering models, have proved questionable at and beyond solid density”*^16^. In this paper, we provide the experimental data and extract the measurement of plasma screening in high energy density regime. We show X-ray emission spectra from copper driven by narrow bandwidth XFEL pulses, with sufficient intensity to heat and ionize the material, generating an array of transitions within the pulse length, including double core hole states. The tunability of the XFEL photon energy is used to resonantly pump transitions with known electronic configuration. By careful analysis of those resonances and comparison to a detailed model of K-shell transitions calculated by the Flexible Atomic Code (FAC)^28^, we connect measured emission lines with the charge state and L-shell occupancy, and consequently measure their shift compared to calculated values. Thanks to relatively new functionality of the FAC code^29^, the atomic calculations can be performed in a plasma environment accounted for by a defined plasma screening model, therefore calculationg Stark shifts. The same transitions are therefore also simulated while applying the screening by the SP model^29^ to show its difference to the experiment. The unique feature of this dataset is that the measurement contains the K$\upalpha$, K$\upbeta$, and K$\upgamma$ transitions, therefore describing the modification of all electronic shells present in the material. The observed shifts are shown to *not* agree well with the Stewart-Pyatt model. The data show the complex structure of the K-shell emission in highly charged ions, and aim to guide the future development and verification of new, more precise, models.

## K-shell transitions in calculations

**Fig. 1:**
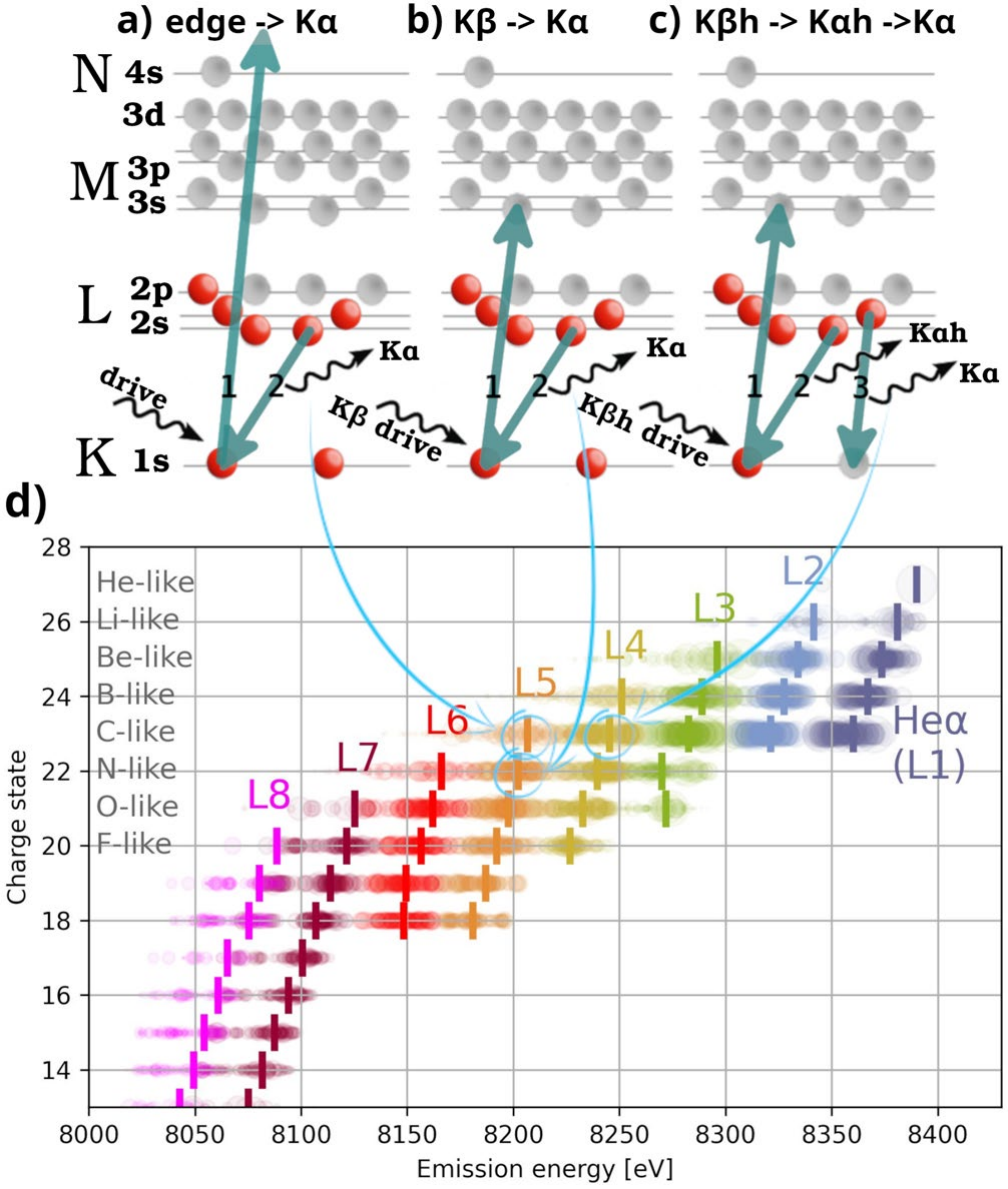
Schematic depiction of observed transition chains: emission above K edge (**a**), K$\upalpha$ emission driven by K$\upbeta$ absorption (**b**), and K$\upalpha$ and K$\upalpha _\textrm{h}$ driven by K$\upbeta _\textrm{h}$ (**c**). K$\upalpha$ transitions calculated by the FAC code (**d**). Each circle is a single transition with size corresponding to its oscillator strength; the transitions are grouped according to charge state (y-axis) and L-shell occupancy (color), and a weighted mean for each group is shown by a vertical marker.

The K-shell energies and oscillator strengths of the emission lines were calculated by the FAC code^28^, details of the calculation are in Supplementary A.7. The $1s^2-2p^n$ transitions are shown in Fig. 1d. A mean energy weighted by the oscillator strength is calculated for each group of transitions given by charge state and L-shell occupancy.

The dominant factor affecting the energy is the occupancy of the L-shell, therefore we label the groups as K$\upalpha$ L*x*, where *x* is the L-shell occupancy in the upper state. Such a description, however, is insufficient to comprehend the full dynamics, as shifts of each of those lines as a function of charge state (or occupancy of M-shell) are resolved. Therefore using a nomenclature K$\upalpha$ L*x* M*y* might be necessary in specific cases. The lines with various M shell occupancies within given K$\upalpha$ L*x* transition are typically unresolvable in experimental data, as their shift is smaller than their widths. In the data shown in this paper, the distinction was made possible by selectively pumping various excitation and ionization states via the K$\upbeta$ transitions, whose sensitivity to M shell occupancy is significantly higher.

Similar data as in Fig. 1d are also calculated for other transitions of interest, namely the K$\upalpha$ transition in so-called hollow ions (ion with a hole in K-shell in the initial state)^30^, further as K$\upalpha _\textrm{h}$  ($1s^1-2p^\textrm{N}$), K$\upbeta$ ($1s^2-3p^\textrm{N}$), K$\upbeta _\textrm{h}$  ($1s^1-3p^\textrm{N}$), and K$\upgamma$ ($1s^2-4p^\textrm{N}$), corresponding figures are shown in Extended data. Further FAC calculations were run with the plasma potential modelled by the SP model, assuming solid density and various plasma temperatures.

In order to demonstrate the basic scaling of those line shifts, an empirical formula is designed to approximate the simulated line positions. This shows that the transition energy can be approximated as

$$\begin{aligned} E = E_0 - k_\textrm{K}K- k_\textrm{L}L - k_\textrm{M}M - c/Te, \end{aligned}$$

where *K*, *L*, and *M* are the occupancies of respective shells, $T_\textrm{e}$ is the plasma electron temperature in eV, and $E_0$, $k_\textrm{K}$, $k_\textrm{L}$,$k_\textrm{M}$, and *c* are constants summarized in Tab.1. The $k_\textrm{x}$ constants indicate how much the emission line shifts with addition of one electron into given shell. Addition of an electron into the L shell introduces a shift of about 44 and 98 eV for K$\upalpha$ and K$\upbeta$, respectively, while the M shell electron causes shifts of only 4, respectively 15 eV. Those shifts are slightly increased for ions with close-to-full L shells. The sensitivity to temperature is significantly higher for K$\upbeta$ compared to the K$\upalpha$ transition. The fit is valid only for charge state discussed in this work, i.e. between 13 and 27 (Table 1).

**Table 1:** Fit parameters for simple formula of K$\upalpha$  and K$\upbeta$  energies.

Case	$E_0$	$k_\textrm{K}$	$k_\textrm{L}$	$k_\textrm{M}$	*c*
K$\upalpha$ ($L\le 6$)	9025	300	44	4	200
K$\upalpha$ ($L\ge 7$)	9025	300	43	6	100
K$\upbeta$ ($L\le 6$)	10,630	380	98	15	500
K$\upbeta$ ($L\ge 7$)	10,630	380	96	19	300
K$\upgamma$	11,110	–	118	30	—

A similar dependence of the transition energy on the presence of electrons in the M-shell (often called spectator electrons) was shown e.g. for Mg He$_\alpha$ in^31^. To our knowledge, however, such shifts were not quantitatively resolved and described before for ions with more than 3 electrons.

### Experiment

**Fig. 2:**
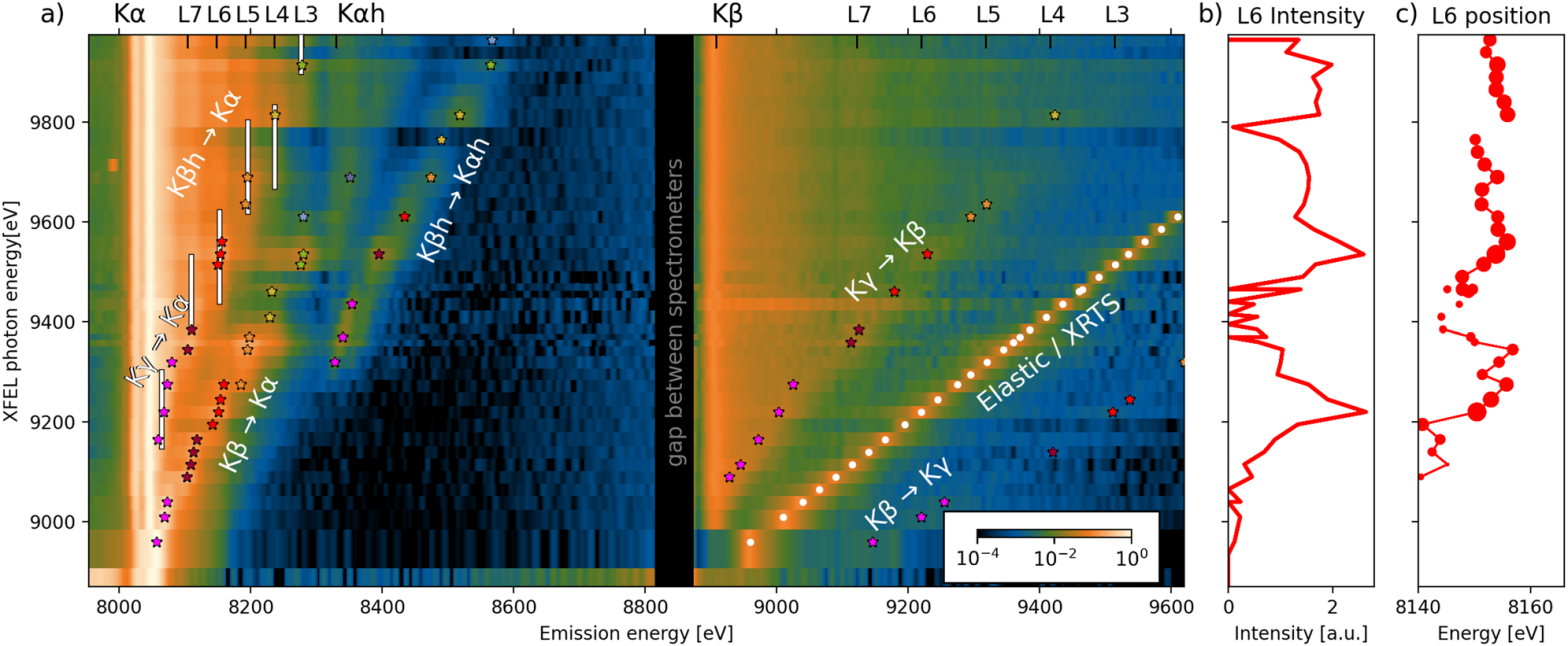
Experimental spectra for beam energy density 110 kJ/cm$^2$(**a**) with identified resonances (stars with color corresponding to L-shell occupancy), edges (white bars), and elastic scattering (white circles). The fitted intensity and position of K$\upalpha$ L6 is shown in (**b**, **c**).

The experiment was performed at the HED instrument of the European XFEL laboratory^32^. The 25 fs long x-ray beam was focused down to a sub-$\upmu$m focal spot reaching intensities up to $7 \times 10^{18}$ w/cm$^2$, corresponding to irradiation of 180 kj/cm$^2$, and its photon energy was varied in the wide range above Cu K edge (8.9–9.9 keV). X-ray emission of the 3 $\upmu$m thick Cu foil was measured by crystal spectrometers covering the range from neutral K$\upalpha$ till highly charged K$\upbeta$ transitions. More details about experiment are in Supplementary section A.1. Spectra were measured for a variable incident pulse energy, and the knowledge of the focal spot distribution (Supplementary A.2) allowed to perform the *focal spot inversion* (Supplementary A.3) to extract the spectra for a given irradiation (areal energy density). The spectra extracted for irradiation of 110 kJ/cm$^2$ and various XFEL photon energies are shown in Fig. 2a. The markers are showing the three key features, which are subject of further analysis: X-ray Thomson scattering (XRTS), resonances, and edges.

### Resonances

The resonant processes mean a chain of two or more electronic transitions, where the first one is driven by photoexcitation, ended with a radiative de-excitation. As the de-excitation follows typically on a few fs timescale (as observed in our simulations), the probability of another process modifying the configuration in between is low in the present cases, therefore we assume the state of the ion is otherwise unchanged. Two such processes are depicted in Fig. 1b,c.

The resonant processes are identified as emission peaks in the spectra for a particular driving energy, whose intensity is decreasing if the driving energy is changed. Measures of the emission were first constructed via fitting a family of Gaussian peaks to the measured spectrum, giving an estimate of yield and position. For some transitions, the precision of the line position was improved in a second by step by fitting the exact line shape. The details of the fit and example of the spectral lineouts are shown in Supplementary methods A.4 and A.5.

The intensity and position of the fits of K$\upalpha$ L6 line are shown in Fig. 2b,c. The two maxima in the intensity plot show the resonant driving via K$\upbeta$  (at $\approx ~9250$ eV) and K$\upbeta _\textrm{h}$  (at $\approx 9500$ eV). In both cases, the emission energy shifts with the change of the driving energy, because different charge states of K$\upbeta$, respectively K$\upbeta _\textrm{h}$, are being pumped, therefore producing K$\upalpha$ emission of ions with corresponding charge states. Once the driving energy moves above $\approx 9600$ eV, both the intensity and position of the line is not changing significantly (apart from jump at $\approx 9780$ eV due to varied experimental conditions at that energy), because it is emitted from ions with K-hole made by photoionization—Fig. 1a—not by photoexcitation.

**Fig. 3:**
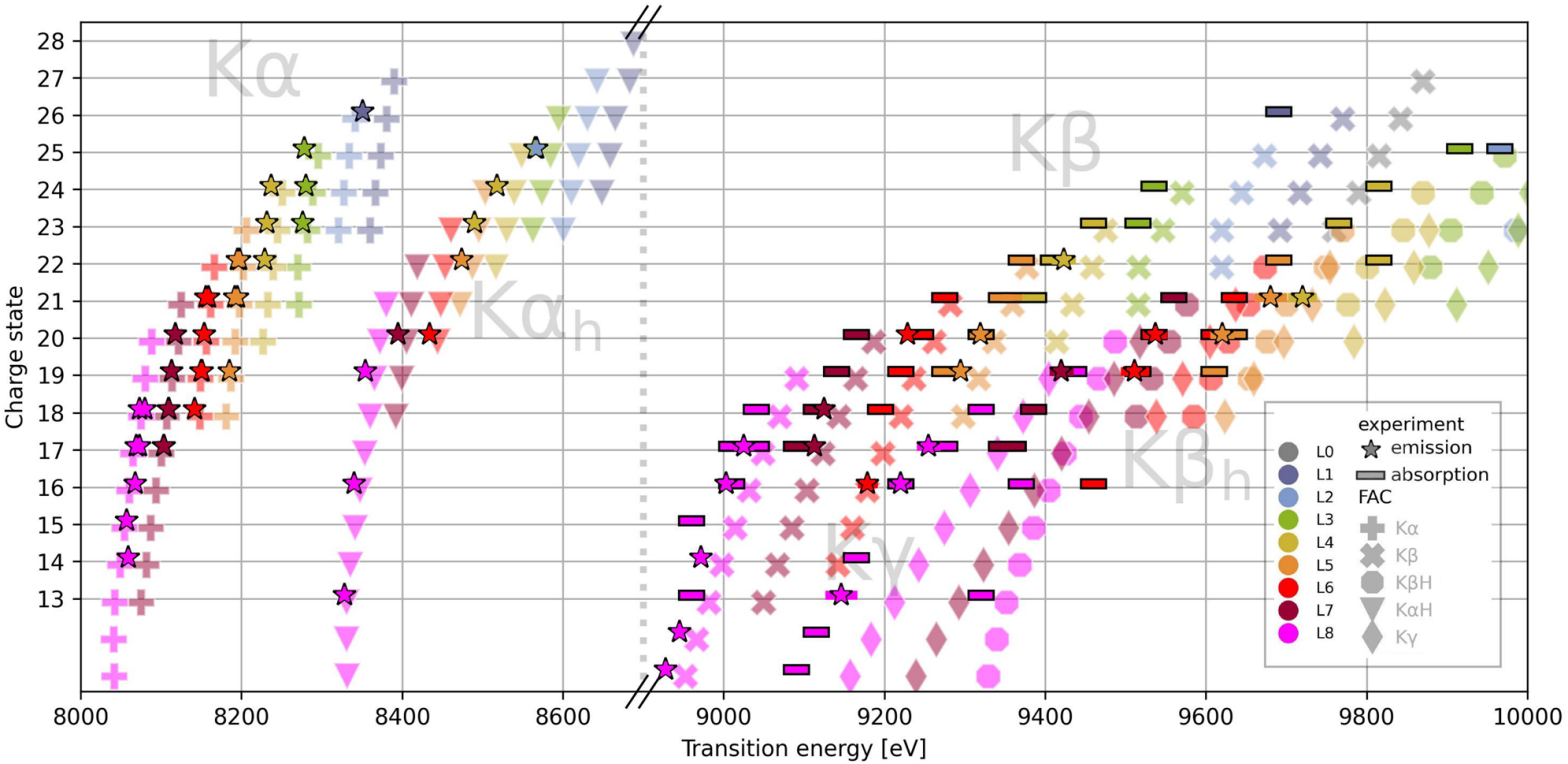
Map of spectral lines in Cu. Black outlined stars show emission lines and bars absorption energies observed in the experiment. Translucent symbols are calculated by the FAC code for isolated atom. Color of symbols corresponds to L shell occupancy.

To identify the electronic configurations of the observed transitions, their energies were compared to the theoretical model, as shown in Fig. 3. Each experimentally observed point from Fig. 2 is interpreted as a pair of ’driving line—emission line’, where the driving line is represented by the horizontal bar (with width of 25 eV showing the uncertainty given by XFEL bandwidth) and the emission by the black-outlined stars. To each measured pair of emission—absorption channels, the charge state and L-shell occupancy is assigned by identifying the theoretical pair with best matching energies. The table with energies identified resonances is presented in Supplementary A.10.

### Edges

Identification of the absorption edges has a long tradition in this type of data^18^, and is typically the easiest measurement that could be done. In copper as well as in other materials with similar Z, however, the edge position shares similar energies as the K$\upbeta _\textrm{h}$ line, undermining the capability to estimate the edge position precisely. Still, we have experimentally identified a relatively broad range of driving photon energies within which the absorption edge can lie, those ranges are shown as white vertical lines in Fig. 2a. The bottom edge of the range is identified so, that if the driving photon energy goes below this edge, the emission of the corresponding K$\upalpha$ line significantly weakens compared to values above it. The upper boundary is the lowest energy above which the emission does not show significant intensity or energy fluctuations. As mentioned, the ranges overlap with the K$\upbeta _\textrm{h}$ – K$\upalpha$ resonances (Fig. 2a), which is the main reason why this data does not allow a more precise estimation of the edge position. The rebinding of states from the continuum, which retain broad energy bands due to the extent of their wavefunction, has been found to make clear identification of an edge position challenging, resulting in different conclusions on the required IPD^19,31,33^.

**Fig. 4:**
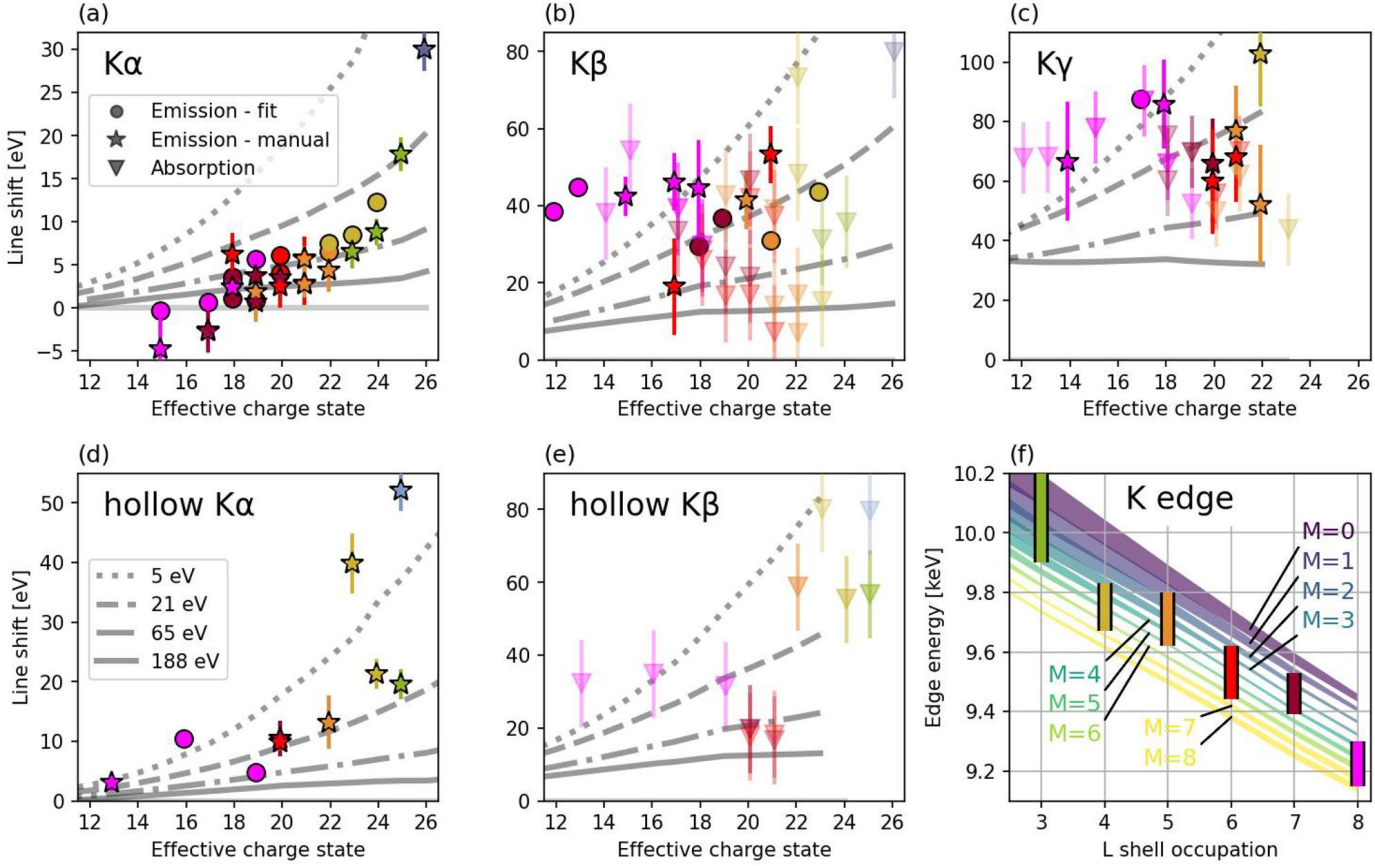
Observed line shifts (**a**–**e**) and edge positions (**f**) for energy density 110 kJ/cm$^2$. Stars and circles are measured by emission, fitted via Gaussian or line-shape, respectively; triangles by absorption with errorbar corresponding to the XFEL bandwidth. The color corresponds to L shell occupancy with same coding as Fig. 3. Grey lines are predictions by SP model in FAC for various temperature assumptions, labeled in (**d**) for all panels. The bands in (**f**) show the calculated edges for various M-shell occupancy, width of band contains data for temperatures between 5 and 107 eV, vertical bars are experimental measurements.

Since the M-shell occupancy and therefore the charge state in the measurement of the K edge is unknown, the resulting data in Fig. 4f are shown as a function of L shell occupancy. The FAC model of the edge position is then plotted by bands for various M-shell occupancies with different colors. Those bands are broad to contain edges for the temperatures between 5 and 107 eV. The effect of temperature is shown to be smaller than the effect of M-shell electrons. The experimental data agree to models with 1 ...7 electrons in M shell. However, the identification of resonant transitions is showing that states with typically between 0 ...4 M shell electrons only are present in the plasma, which would produce smaller shift of the edge energy. Therefore, the edge measurement also indicates the screening and CL is stronger than predicted by SP model.

### Charge-state dependence of plasma screening

In this work, plasma screening is measured as a difference between the observed transition energy and calculation of its energy for isolated atom. The found values are plotted in Fig. 4 for each transition separately as a function of effective charge state. Effective charge state in this context is charge state calculated from occupancy of K, L and M shells, i.e. ignoring electrons in N shell, which have negligible effect on screening, but in studied case appeared due to K$\upgamma$ excitation. Plasma screening calculated by the FAC code using the Stewart-Pyatt model is shown in grey lines, assuming solid density and different plasma temperatures.

The trend that plasma screening is increasing with charge state agrees. However, it is not following the trend lines and the models would have to assume lower temperatures (between 5 and 20 eV), which is significantly lower than the expected values of more than a hundred eV, similar to other x-ray isochoric heating investigations for similar states^31^.

The K edge is shown in Fig. 4f. Each band shows variation of edge position for the temperatures 5 ...188 eV, showing that the temperature effect is negligible. On the other hand, the effect of M-shell occupancy is strong, each electron in M band decreasing the edge by 50 eV. We can therefore conclude that the experiment agrees to model only if M-shell occupation is between 2 and 6 electrons for L=4...7, and more than 5 electrons for L=8. Those numbers are not that unreasonable, but from the identification of resonances, we expect less electrons in M shell, therefore indicating that the modelled shift is underestimated.

### Thermal conditions

**Fig. 5:**
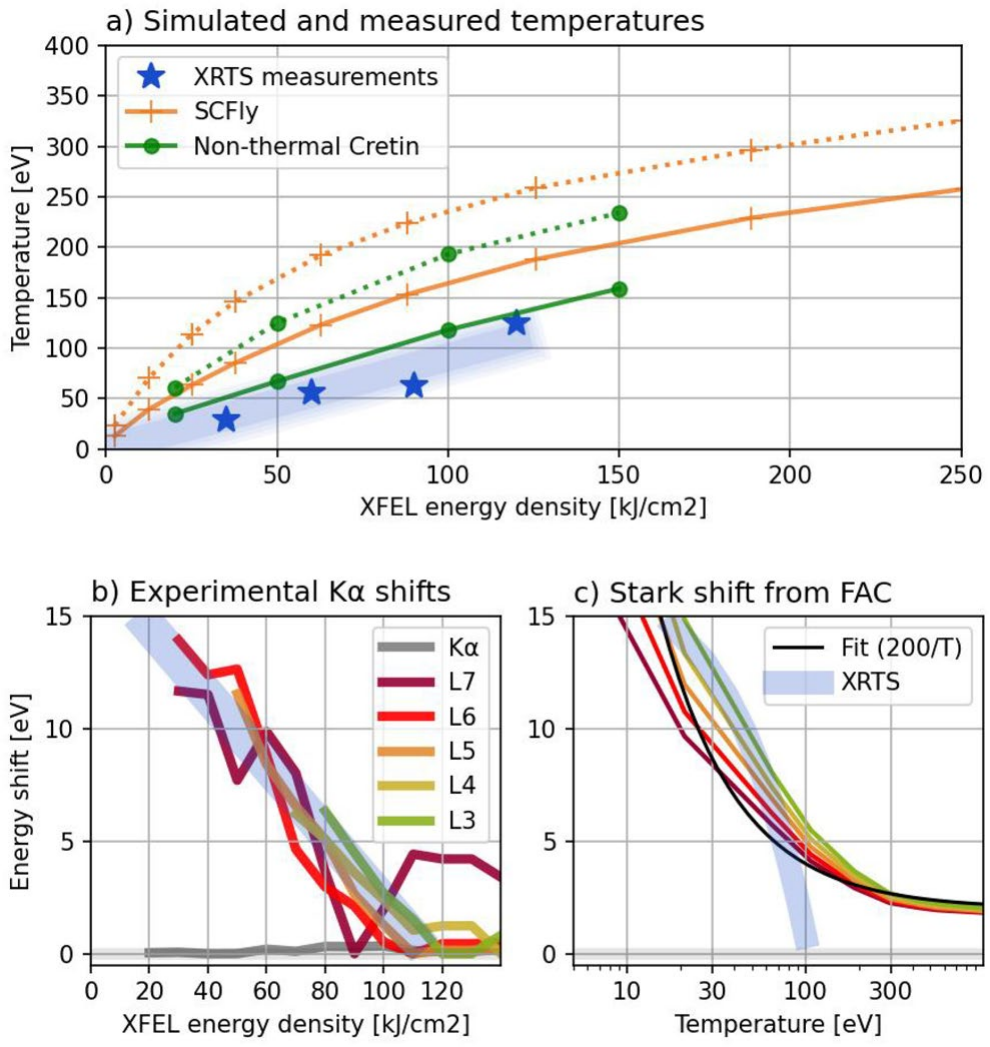
Temperatures of the plasma calculated by the CR codes SCFLY and Cretin (**a**). Solid lines are temperatures during the peak of the XFEL beam, dotted lines are the maximal temperatures, reached toward the end of the pulse. Stars indicate temperatures estimated from the XRTS data. Observed (**b**) and predicted (**c**) Stark shifts as a function of energy density and plasma temperature, respectively. Blue band in (**b**) is a linear fit to the data, and is transferred into (**c**) by using the measured temperature-energy density relation in (**a**). Black fit in (**c**) indicate scaling used in the empirical formula.

There are several ways to assess the thermal conditions in the target. First, a set of simulations with the SCFLY collisional radiative (CR) code^34^ was performed (Supplementary A.8). The inherent disadvantage of that code is that it assumes thermal distribution of electrons, which in general might not be true, as the dominant mechanism of energy absorption is photoionization, producing electrons with very non-thermal energies. However, as shown in^35^ and confirmed by our simulations with the non-thermal version of the code Cretin (Supplementary A.9), the electron distribution in this case is close to Maxwellian due to rapid thermalization via frequent electron–electron collisions, allowing the temperature to be used as a suitable metric. The Cretin code was run with comparable conditions, and the temperatures obtained from both codes are presented in Fig. 5a.

The experimental approach analyses the elastic scattering data (XRTS) using the approach shown in^36^, see Supplementary A.6. The temporal integration of the signal, overlap of XRTS with emission, and overall signal to noise ratio, however, limits its accuracy. The results are shown in Fig. 5a and are in good agreement to the simulated values for temperatures during the peak irradiation. For energy density 110 kJ/cm$^2$, the measured plasma temperature is $T \approx 100$ eV, while the Cretin code predicts 115 eV during the peak of XFEL beam.

### Temperature dependence of plasma screening

All experimental spectra shown until this point were obtained with an irradiation of 110 kJ/cm$^2$. Investigations of line positions from different heating conditions can reveal the Stark shifts as a function of temperature. The shifts of the K$\upalpha$ Lx emission extracted from spectra with various XFEL energy densities is shown in Fig. 5b. All transitions show a very similar trend—about 13 eV shift between 40 and 120 kJ/cm$^2$. Such shift corresponds to the theoretical model of plasma screening (Fig. 5c) with temperature of about 20 eV. Note, that in contrast to common intuition, the higher temperatures correspond to more equilibrated conditions in this situation: We observe lines with charge state 22 or more, which would be present in equilibrated plasmas only at much higher temperatures (one or a few keV); The lower the temperature we observe those transitions at, the further the conditions are from any kind of equilibria, and the stronger the Stark shift is.

Having a reliable Stark shift model for those conditions, those shifts could be used to infer the electron temperature of the plasma. Yet, an inverse process can be applied here: Observed line shifts are ascribed to plasma temperature from the XRTS measurements, and therefore an empirical curve of Stark shift as a function of temperature is plotted in Fig. 5c with broad blue line. This shows reasonable agreement with the theoretical prediction.

### Summary

We have mapped the K shell transitions in intermediate to highly charged matter by using resonant pumping by the high intensity XFEL beam. The connection between the absorbing and emitting line allowed identification of the L and M shell occupancy, and therefore resolution of the transitions with unprecedented details, i.e. to distinguish its emission for various occupancies of K, L and M shells. The FAC atomic calculations of those transitions in isolated ions, as well as with plasma screening by the Stewart-Pyatt model with various plasma temperature assumptions were compared to the experimental observation to identify the discrepancies. In both the charge-state and temperature dependent measurement, experimental data are found to be matched only by the SP model when assuming unreasonably low temperatures—reconfirming the observation that this model systematically underestimates the plasma screening in this regime. This statement was so far observed only for charge states below 15, our data confirms it for charge state up to 26. The presented experimental measurements of shifts of K$\upalpha$, K$\upbeta$, K$\upgamma$ transitions and their hollow partners provides a complex information about the modification of the ionic electronic potential in a well defined plasma environment. These measurements shall stimulate the development and verification of novel codes to model the potential and improve our understanding of precise atomic physics in Warm Dense Matter.

## Supplementary Information

Supplementary Information.

## Data Availability

The raw data are published by the European XFEL laboratory (https://in.xfel.eu/metadata/doi/10.22003/XFEL.EU-DATA-002806-00), the processed data (https://doi.org/10.14278/rodare.2789) and the analysis scripts (https://doi.org/10.14278/rodare.2791) are published in the Rodare repository.
