# Supplementary material for: Plasma screening in mid-charged ions observed by K-shell line emission
Source: Sci Rep. 2026 Feb 10;16:5873. doi: 10.1038/s41598-026-39041-1 (PMC12894718; doi:10.1038/s41598-026-39041-1)
Supplement: Supplementary file 1 — Supplementary Information. [file 41598_2026_39041_MOESM1_ESM.pdf]

# Supplementary information
# Plasma screening in mid-charged ions observed by K-shell line emission

M. Šmíd,[1,*] O. S. Humphries,[2] C. Baehtz,[1] V. Bouffetier,[2,1] E. Brambrink,[2] T. Burian,[3] V. Cerantola,[2,4] M. S. Cho,[5] T. E. Cowan,[1] L. Gaus,[1] M. F. Gu,[6] V. Hájková,[3] L. Juha,[3] J. Kaa,[2] Z. Konopkova,[2] M. Kozlová,[7,8] H. P. Le,[5] M. Makita,[2] X. Pan,[1,9] T. R. Preston,[2] A. Schropp,[10] J.-P. Schwinkendorf,[1] H. A. Scott,[5] R. Štefaníková,[1] J. Vorberger,[1] W. Wang,[10] U. Zastrau,[2] and K. Falk[1,3]

[1] *Helmholtz Zentrum Dresden Rossendorf, Bautzner Landstraße 400, 01328 Dresden, Germany*
[2] *European XFEL, Holzkoppel 4, 22869 Schenefeld, Germany*
[3] *Institute of Physics, Czech Academy of Sciences, Na Slovance 2, 182 00 Praha, Czech Republic*
[4] *Department of Earth and Environmental Sciences, University of Milano-Bicocca, Piazza della Scienza 4, 20126 Milano, Italy*
[5] *Lawrence Livermore National Laboratory, 7000 East Avenue, Livermore, California 94550, USA*
[6] *Space Science Laboratory, University of California, Berkeley, California 94720, USA*
[7] *Institute of Plasma Physics of the Czech Academy of Sciences, U Slovanky 2525/1a,182 00 Praha, Czech Republic*
[8] *The Extreme Light Infrastructure ERIC, ELI Beamlines Facility, Za Radnici 835,, 25241 Dolni Brezany, Czech Republic*
[9] *Technische Universität Dresden, 01062 Dresden, Germany*
[10] *Centre for X-ray and Nano Science CXNS, Deutsches Elektronen-Synchrotron DESY, Notkestrasse 85, 22607 Hamburg, Germany*

(Dated: January 26, 2026)

## A.1 Experimental details

The XFEL beam was operated in the SASE regime and was focused with a stack of 20 Beryllium lenses with radius of curvature 50 μm, providing a focal length of ≈ 30 - 40 cm, based on the XFEL photon energy. The bandwidth was measured via scattering on a cold sample and had a FWHM about 15 eV. The photon energy was scanned in the range 8750 - 9900 eV with approximately 25 eV steps. The x-ray emission was measured by three crystal spectrometers. Two of them employed the HAPG crystal and were aligned to the range 7900 - 8800 eV (measuring at scattering angle 35° - forward) and 8950 - 9750 eV (scattering angle 170° - backward), respectively [1]. The third one employed a Germanium crystal and observed the details of Cu Kβ emission in the range 8950 - 9400 eV with higher resolution [2]. The spectrometers were initially calibrated by measuring the emission of non-heated lines of Cu, Zn and Ni. However, during data analysis it was found the calibration of the HAPG spectrometer was shifting throughout the experiment, most likely due to an unknown mechanical issue. This is shown in Fig. 1, where the results of the fits of Cu Kα and Kβ are shown as a function of run number – i.e. during the experimental progress, approximately 2 days. This shift was then fitted (straight lines) and all data were corrected by the found offset. The accuracy of spectral calibration is therefore better than 3 eV.

The energy of the XFEL beam was measured by fitting the XRTS peak visible in the spectrometers. The precision of the measurement is limited by the width of the SASE spectrum and spectrometer calibration, but can be estimated as better than 5 eV.

The target consists of simple foils held in a 6 mm × 30 mm window, allowing a continuous shooting with repetition frequency 10 Hz, when the speed of the target holder was adjusted to keep the spacing between shots 20 μm. The data in this paper are from shots where target was a 3 μm thick Cu foil.

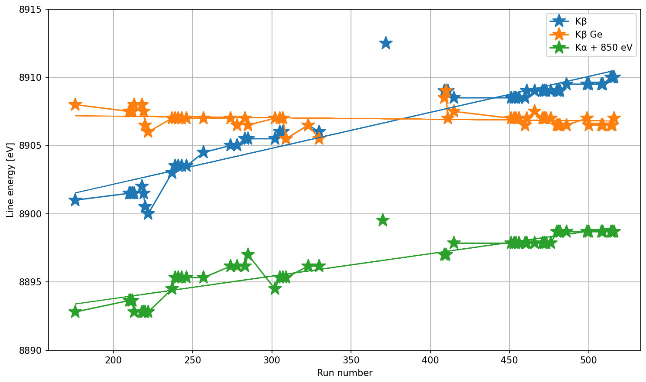


FIG. 1. Calibration of spectrometers. Fitted position of Kα or Kβ on the three used spectrometers as a function of run number, showing its shift during the progress of the experiment. The shown linear fit was used to correct for this shift. (Figure will be yet visually improved).

## A.2 Focusing

The focal length of the lenses is changing due to its chromacity by approximately 1 mm per 25 eV. Therefore, the focusing had to be optimized after each change of energy. This was done via continuous data acquisition while the XFEL was on and the lens position was changing. From such data, the position where strongest emission of ionized Kα lines was observed was identified as the focused one. The quality and characteristics of the focus was analyzed by the imprinting technique [3] at three photon energies (8900, 9400, and 9900 eV). The analysis of the imprints provided the encircled energy curves, Fig. 2.

* m.smid@hzdr.de

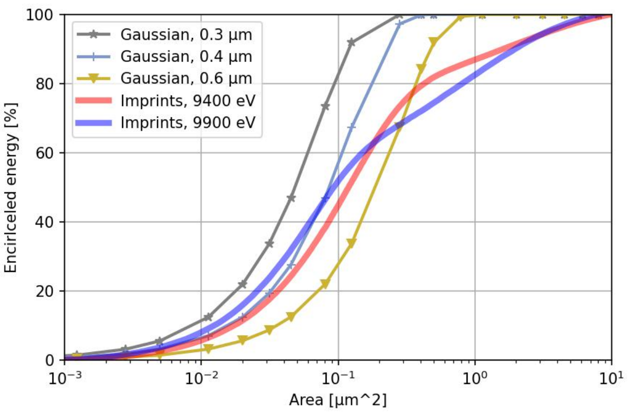


FIG. 2. Focal spot characteristics (encircled energy) from the imprint measurement. Thin lines are calculations of 2D Gaussian spots for comparison.

At all three cases it had shown comparable results, that the distribution of inner 50% of energy was resembling 0.4 μm diameter Gaussian spot. Such size of the focus was mainly limited by the bandwidth of the beam.

### A.3 Beam energy and intensity calibration

The beam energy on the target was measured by a diode detector coupled to a diamond screen, located between the last focusing element and the target. This detector was absolutely calibrated with the x-ray gas monitors. The last lens has an aperture of 300 μm, and was intentionally overfilled, i.e. the beam size on its entrance was kept in the range of approx. 350 - 500 μm. This overfilling converted the spatial jitter of the XFEL beam into an energy jitter, which means that in each data run, there was a large fluctuation of energy in individual shots, ranging between 30 to 280 μJ. Each data run consists of typically 3000 shots, with nominally identical conditions. Due to the energy jitter, those shots have been grouped based on the measured energy providing energy resolved spectra.

On those data, the so called *focal spot inversion* was performed. This relies on the fact that experimental spectra for each beam energy, $S_E$, are integration of constituent spectra from various intensities, with ratios given by the focal spot distribution $f_I$,

$$S_E(E) = \int S_I(I) f_I(I) dI,$$

where $I$ is the XFEL beam intensity, and $S_I$ is the emitted spectrum for given intensity. When considering a Gaussian focal spot profile,

$$I(r) = I_0 \exp\left(-\frac{r^2}{2\sigma^2}\right), \tag{1}$$

and considering the area enclosing each intensity contour $A = \pi r^2$, it is possible to construct the areal density

$$I(A) = I_0 e^{-\frac{A}{2\pi\sigma^2}} \tag{2}$$

$$A(I) = \begin{cases} 2\pi\sigma^2 \ln(I_0/I) & 0 < I < I_0 \\ 0 & \text{otherwise} \end{cases} \tag{3}$$

$$\frac{dA}{dI} = \begin{cases} -\frac{2\pi\sigma^2}{I} & 0 < I < I_0 \\ 0 & \text{otherwise} \end{cases} \tag{4}$$

$$\tag{5}$$

from which it is possible to construct the inverse mapping

$$S_E(E) = \int_0^{I_0} S_I(I) \left|\frac{dA}{dI}\right| dI \tag{6}$$

$$= \int_0^{I_0} \frac{2\pi\sigma^2 S_I(I)}{I} dI \tag{7}$$

$$I_0 = \frac{E}{f} 2\pi\sigma^2 \tag{8}$$

$$\frac{dS_E(E)}{dE} = \frac{S_I(I)}{I}. \tag{9}$$

The discretized form of the differential was used to obtain the spectra presented in this paper

$$S_I(I)/I = \frac{dS_E}{dE},$$

where we retain the normalization by $I$ to yield the emitted spectrum for a given incident power at the specified intensity, so that line emission intensity is comparable.

While, as shown in Fig. 2, the focal spot is not purely Gaussian, the most intense parts of the distribution – which is responsible for the heated emission – is well described by a Gaussian profile. This could therefore be expected to induce errors on reconstructed emission from ground state emission lines, Kα and Kβ, which have contributions from the large wings of the focal spot profile – however does not affect the conclusions of this work on the plasma screening within solid density HED plasmas.

### A.4 Gaussian fit of spectra

The measured spectra were first fitted with a set of Gaussian curves. The fit minimizes the least squares of differences between the experimental spectrum and fitted curve, but due to a high non-linearity of the problem, a custom algorithm was applied.

In order to catch the properties of very low intensity lines comparably well to the strong ones, the fit is performed in the *log* space. The algorithm is iterative. For the Kα spectra, it is fitting sum of 11 Gaussian lines. In order to gain convergence, appropriate initial conditions have to be set. The initial line positions are $8027, 8047, 8055, 8065, 8110, 8150, 8195, 8235, 8278, 8332$ and 8355 eV, the initial widths are 15 eV for the cold Kα, and 35 eV for the satellites, the amplitudes are set to correspond to experimental values at the positions. The fit is then minimizing the difference in the range $8010 - 8400$ eV. The components with initial conditions

between 8065 eV and 8278 eV corresponds to the L8 – L3 transitions. Components with higher initial energy fill the $K\alpha_h$ range, which is then fitted separately. The spectra, initial conditions, and fit results including separate components are shown in Fig. 3.

The same algorithm is then used to fit the $K\beta$ and $K\alpha_h$ range. For $K\beta$, the range 8800 – 9600 eV is fitted, and the components are starting at $8905, 8920, 8980, 9050, 9120, 9200, 9300, 9400$ and $9520$ eV. For $K\alpha_h$, the fitted range is 8300 – 8600 eV and the initial conditions are $8330, 8348, 8440, 8480$ and $8650$ eV. The spectra and results are shown in Fig. 4

This approach is numerically well reliable, but lacks the details of shapes of specific lines. Therefore a second step, described in next section, uses the additional information to improve the precision of line shift measurement.

### A.5 Fine-shape fit of spectra

Measuring the shift of each line as a difference of theoretical and experimental position can be misleading due to often complicated line shapes. As, for example, the essential $K\alpha$ is formed by a doublet with $\approx 20$ eV spacing. In the theoretical data, the weaker component is seen and affects the mean (central) position of the peak, while in complex experimental spectra might be neglected or confused with emission of neighboring charge states.

To overcome this, we have developed following approach to fit the shift of each component by using the complex line shape as predicted by the simulation. This process is performed for each identified transition separately (further denoted as *selected component*), and is illustrated in Fig. 5 on example of $K\alpha$ in Z=19 with 8 electrons in L shell. First, the fit of experimental spectra by a set of Gaussian components is taken, as decribed in previous section (dotted line). The Gaussian component corresponding to the *selected* line is then removed and replaced by the spectrum of given line, as calculated by the FAC code (blue line). The shift, amplitude, and broadening of this component is fitted via least square method to get best agreement (black line) to the experimental curve.

The chosen example shows that the stronger, higher-energy component of the transition (8080 - 8090 eV) is well fit to the shoulder on experimental data at 8080 eV, while the other, low energy part of spectra, is still seen in the data at 8060 eV. This fit result in shift of 6 eV of this component. This and other values where this fit worked properly are shown in Fig. **??**(a) by stars, instead of the general Gaussian fit shown by circles.

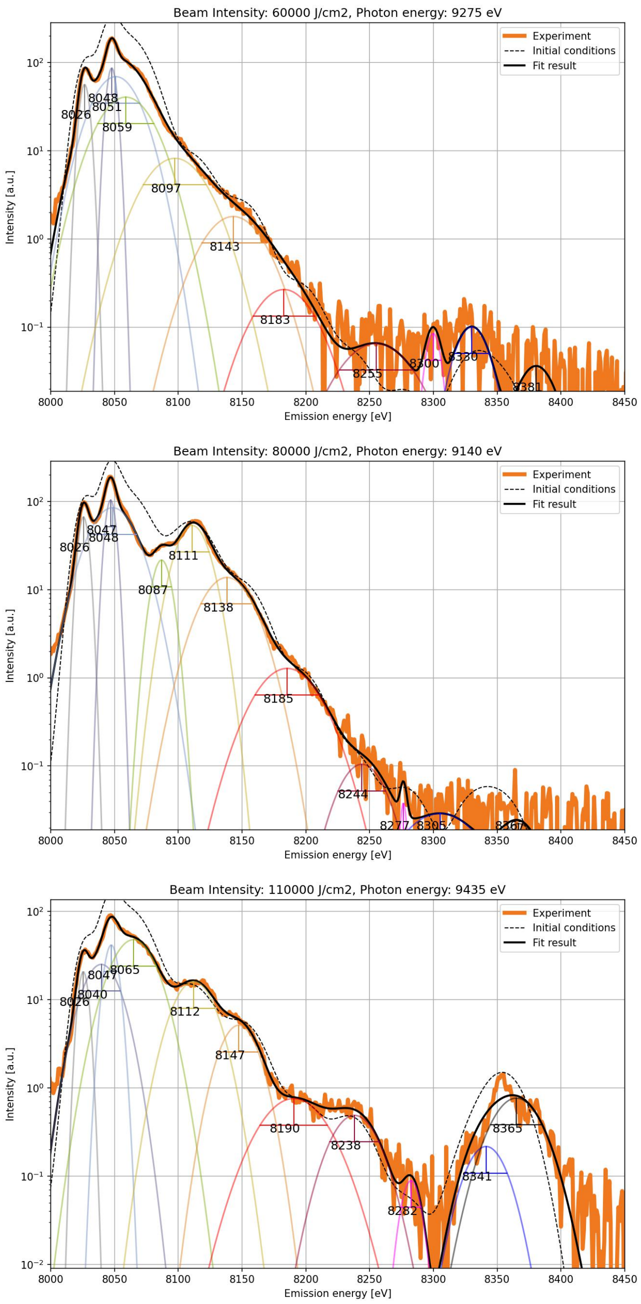


FIG. 3. Selected experimental spectra in the $K\alpha$ range and their fits for few selected irradiation conditions, see respective titles. The colored parabolas depicts the Gaussian components of the fits with label stating fitted peak energy.

### A.6 XRTS

The XRTS spectrum measured at scattering angle 170° (see Fig. 6), was analysed by the imaginary time thermometry method [4, 5]. The XRTS spectrum and the source and instrument function are subjected to a two-sided Laplace transformation. The resulting quotient of the two quantities is symmetric in imaginary time $\tau$-space around the inverse temperature $k_B T/2$ due to detailed balance. This allows the temperature to be extracted in a model-free manner.

### A.7 Atomic simulations

The FAC code was used to calculate the energies of x-ray lines and edges. In this case, we have very high control of the atomic model, and using the new functionality of FAC [6], we can vary the plasma screening model and see

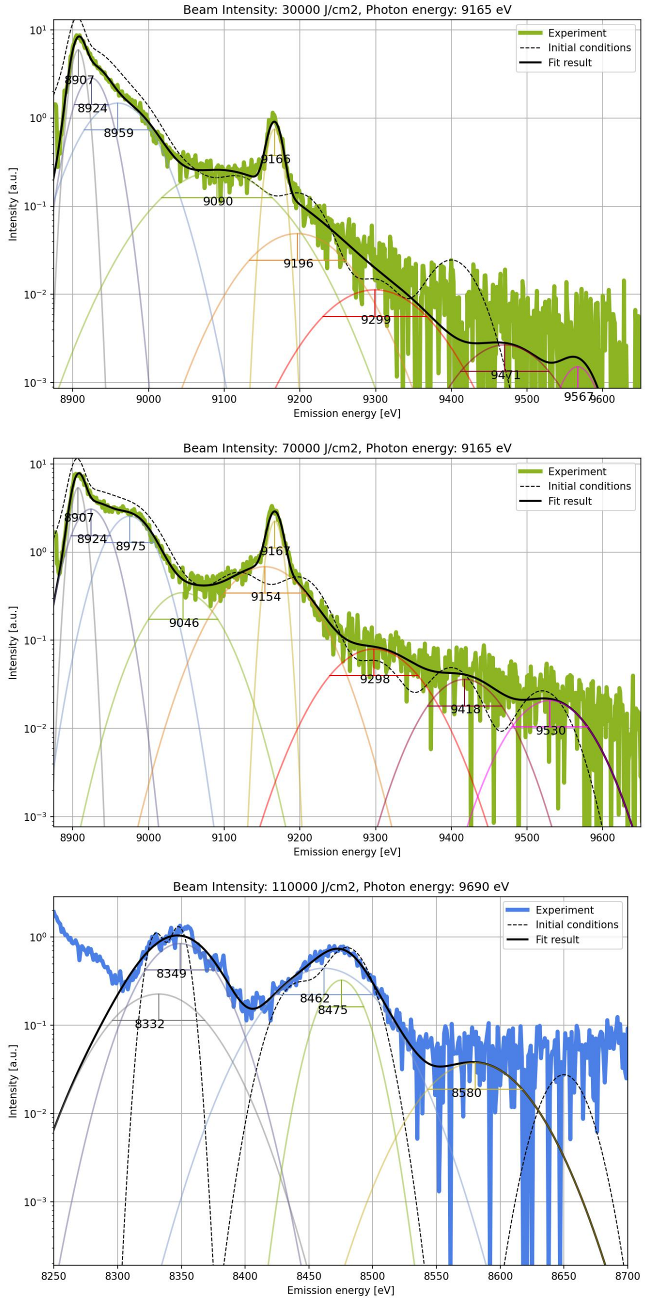


FIG. 4. Experimental spectra in the Kβ and $K\alpha_h$ range and their fits for few selected irradiation conditions, see respective titles. The colored parabolas depicts the Gaussian components of the fits with label stating fitted peak energy.

its effect on those (the Stark shift). Results of those calculations are presented in Fig. ??(c) and described in the main text. The input files for the model are generated by a script which looks for levels needed to produce the desired transitions. There are typically tens of configurations per charge state, the whole model then has about 226 000 levels.

The accuracy of line position calculation in isolated ion

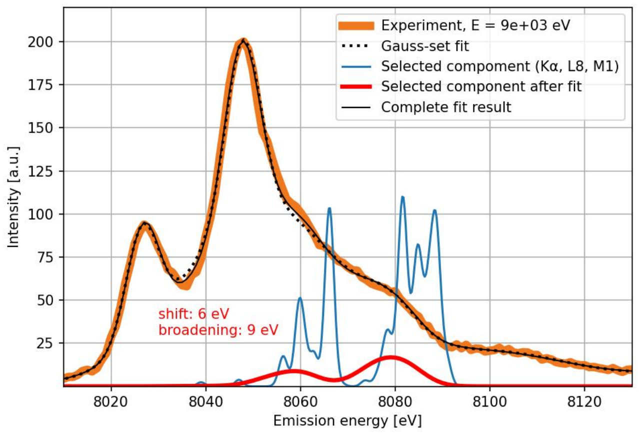


FIG. 5. Illustration of fitting of shift of single component.

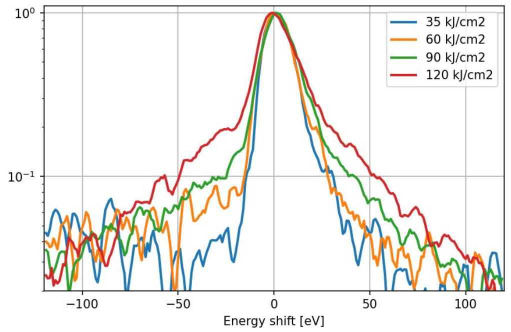


FIG. 6. Spectra used for the XRTS analysis

is critical for the analysis in this paper. The accuracy of FAC calculations on Si and S was assessed in [7], showing a very good agreement on a 2-3 eV level. In Cu, the EBIT measurements were compared to the second order many-body perturbation theory (MBPT mode) FAC calculations yielding typically 0.5 eV accuracy [8]. That is however a more involved mode, which may not be possible for very complex ions with many open M-shell elec-

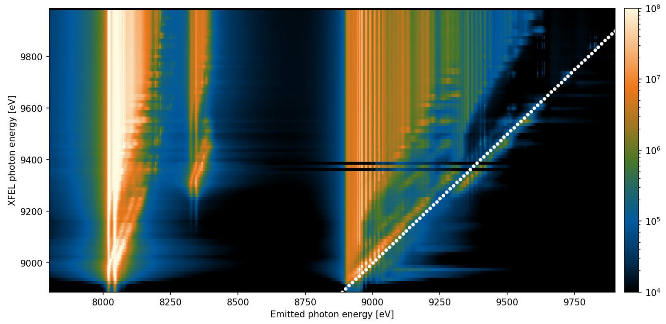


FIG. 7. Spectra simulated by the SCFLY code for irradiation 125 kJ/cm$^2$.

trons as are used in this work. Calculations in this paper are therefore done in the configuration interaction (CI) mode only. To benchmark those, a simulations in identical settings but on Fe were performed and compared to the experimental data presented in [8]. The results are shown in Tab. I, showing a $< 2$ eV accuracy. This is much smaller than scale of line shift discussed in this paper (tens eV).

| Element | Line | $E_{\mathrm{exp}}$ [eV] | $E_{\mathrm{FAC}}$ [eV] | $\Delta$ [eV] |
|---|---|---|---|---|
| Fe XXII | C1 | 6544.2 | 6542.8 | 1.4 |
| Fe XXII | C2 | 6556.9 | 6554.5 | 2.4 |

TABLE I. Comparison of line positions from our atomic calculations ($E_{\mathrm{FAC}}$) to experimental values presented in Table 1 of [8] ($E_{\mathrm{exp}}$) and their difference.

Figure 8 shows calculations for further lines with no plasma screening. Figure 9 shows calculations for K$\alpha$ line with plasma screening following the Stewart-Pyatt model for various assumptions of electron temperature.

### A.8 Collisional radiative simulations

The SCFLY code was used to model the interaction in a 0D, time-dependent collisional-radiative (CR) simulations with zero initial temperature, and with heating by XFEL beam with super Gaussian temporal profile. We have performed a set of simulations with various beam and photon energies, to simulate the whole set of experimental data, see Fig. 7. We can observe the simulation matches the experiment qualitatively very well, but such approach is not suitable for quantitative analysis as the atomic model lacks the necessary details.

### A.9 Cretin non-thermal CR simulations

The Cretin simulations model the evolution of the electron distribution and do not assume a thermal distribution. It evolves the electron energy distribution self-consistently with the atomic populations. The electron distribution evolution is governed by a kinetic Boltzmann-Fokker-Planck equation which includes all elastic (electron – electron collision) and inelastic collisional and radiative processes [9]. The atomic model used in the Cretin simulations has the same set of energy levels and transitions as SCFly. Since the model does not assume thermal distribution, the temperature shown in Fig. ??(a) is defined as 2/3 of the kinetic energy of the electrons. Deviation from a thermal distribution can be calculated using the non-equilibrium factor defined by Hau-Riege [10]. First, the energy is decomposed into a low-energy and a high energy component. The non-equilibrium factor is defined as the ratio of the kinetic energy of the high energy component relative to the total kinetic energy, i.e., 0 means thermalized distribution and 1 means complete non-equilibrium distribution. This factor is decreasing below 0.1 towards the end of the XFEL pulse, showing the plasma is sufficiently thermalized towards the end of the pulse.

This is in agreement with theoretical electron–electorn relaxation time, which is calculated using a Fermi-liquid estimate for solid-density copper at $T_e = 100\,\mathrm{eV}$, assuming an average ionization $\bar{Z} \sim 10$, on the order of $\tau_{ee} \sim 5$–$10\,\mathrm{fs}$, confirming that the considered conditions are just on the boundary of electronic thermalization.

Similar non-thermal simulations were performed in [11], concluding that *"non-thermal electrons become important in determining the short-time plasma kinetics and electron dynamics for intensities above* $10^{17}\,W/cm^2$*, and for pulse lengths below 10 fs"*. That also confirms that our case, with almost twice as long driving pulse length is closer towards the thermalized case.

### A.10 Resonances

The identified resonances are written as the pairs of driving (absorption) and emission energy in Tab.II. The uncertainty of emission energy is written in table, the absorption uncertainty is given by the 25 eV bandwidth of the XFEL. Second section of the table assign identified processes and charge state to the pair. Last column classifies certainty of this assignment: 3 - confident identification, 2 - less confident identification, 1 - estimate, 0 - no suitable identification was found. Last section shows the result of shift fitting using the FAC line shape, as discused above. The fitted shift and broadeding is stated.

| # | Energy [eV] | | | Identification | | | | | Fit [eV] | |
|---|---|---|---|---|---|---|---|---|---|---|
| | Absorption | Emission | Unc. | Z | L | Process | States | Cert. | Shift | FWHM |
| 5 | 9165 | 8059 | 2.5 | 14 | 8 | G - A | K2 L8 M5 - K1 L8 M5 N1 - K2 L7 M5 N1 | 1 | | |
| 8 | 9220 | 8068 | 2.5 | 16 | 8 | G - A | K2 L8 M3 - K1 L8 M3 N1 - K2 L7 M3 N1 | 2 | | |
| 9 | 9010 | 8069 | 1.5 | 17 | 8 | B - A | K2 L8 M2 - K1 L8 M3 - K2 L7 M3 | 2 | 1 | 14 |
| 11 | 9040 | 8073 | 1.5 | 18 | 8 | B - A | K2 L8 M1 - K1 L8 M2 - K2 L7 M2 | 2 | | |
| 12 | 9275 | 8073 | 2.5 | 17 | 8 | G - A | K2 L8 M2 - K1 L8 M2 N1 - K2 L7 M2 N1 | 3 | 3 | 12 |
| 15 | 9320 | 8080 | 1.5 | 18 | 8 | G - A | K2 L8 M1 - K1 L8 M1 N1 - K2 L7 M1 N1 | 2 | 6 | 9 |
| 19 | 9090 | 8928 | 5.0 | 11 | 8 | G - B | K2 L8 M8 - K1 L8 M8 N1 - K2 L8 M7 N1 | 2 | 39 | 45 |
| 20 | 9115 | 8945 | 5.0 | 12 | 8 | G - B | K2 L8 M7 - K1 L8 M7 N1 - K2 L8 M6 N1 | 1 | 45 | 49 |
| 22 | 9165 | 8972 | 5.0 | 14 | 8 | G - B | K2 L8 M5 - K1 L8 M5 N1 - K2 L8 M4 N1 | 1 | | |
| 25 | 9220 | 9003 | 7.5 | 16 | 8 | G - B | K2 L8 M3 - K1 L8 M3 N1 - K2 L8 M2 N1 | 2 | | |
| 27 | 9275 | 9025 | 12.5 | 17 | 8 | G - B | K2 L8 M2 - K1 L8 M2 N1 - K2 L8 M1 N1 | 3 | | |
| 29 | 8960 | 9146 | 20.0 | 13 | 8 | B - G | K2 L8 M5 N1 - K1 L8 M6 N1 - K2 L8 M6 | 1 | | |
| 30 | 9010 | 9220 | 10.0 | 16 | 8 | B - G | K2 L8 M2 N1 - K1 L8 M3 N1 - K2 L8 M3 | 2 | 88 | 86 |
| 31 | 9040 | 9255 | 15.0 | 17 | 8 | B - G | K2 L8 M1 N1 - K1 L8 M2 N1 - K2 L8 M2 | 3 | | |

| | | | | | | | | | | |
|---|---|---|---|---|---|---|---|---|---|---|
| 33 | 9320 | 8328 | 1.5 | 13 | 8 | Bh - Ah | K1 L8 M7 - K0 L8 M8 - K1 L7 M8 | 1 | | |
| 34 | 9370 | 8340 | 1.5 | 16 | 8 | Bh - Ah | K1 L8 M4 - K0 L8 M5 - K1 L7 M5 | 1 | 11 | 64 |
| 35 | 9435 | 8354 | 1.0 | 19 | 8 | Bh - Ah | K1 L8 M1 - K0 L8 M2 - K1 L7 M2 | 1 | 5 | 36 |
| 40 | 9090 | 8103 | 2.5 | 17 | 7 | B - A | K2 L7 M3 - K1 L7 M4 - K2 L6 M4 | 2 | | |
| 41 | 9345 | 8104 | 1.5 | 17 | 7 | G - A | K2 L7 M3 - K1 L7 M3 N1 - K2 L6 M3 N1 | 2 | 4 | 29 |
| 42 | 9115 | 8109 | 2.5 | 18 | 7 | B - A | K2 L7 M2 - K1 L7 M3 - K2 L6 M3 | 3 | 1 | 32 |
| 43 | 9385 | 8110 | 1.5 | 18 | 7 | G - A | K2 L7 M2 - K1 L7 M2 N1 - K2 L6 M2 N1 | 2 | | |
| 45 | 9140 | 8113 | 1.5 | 19 | 7 | B - A | K2 L7 M1 - K1 L7 M2 - K2 L6 M2 | 3 | | |
| 46 | 9165 | 8118 | 1.5 | 20 | 7 | B - A | K2 L7 M0 - K1 L7 M1 - K2 L6 M1 | 3 | | |
| 47 | 9535 | 8154 | 2.5 | 20 | 6 | Bh - A | K1 L7 M1 - K0 L7 M2 - K1 L6 M2 - K2 L5 M2 | 3 | | |
| 48 | 9560 | 8156 | 2.5 | 21 | 6 | Bh - A | K1 L7 M0 - K0 L7 M1 - K1 L6 M1 - K2 L5 M1 | 1 | | |
| 49 | 9535 | 8395 | 3.0 | 20 | 7 | Bh - Ah | K1 L7 M1 - K0 L7 M2 - K1 L6 M2 | 2 | | |
| 52 | 9360 | 9113 | 5.0 | 17 | 7 | G - B | K2 L7 M3 - K1 L7 M3 N1 - K2 L7 M2 N1 | 3 | 30 | 62 |
| 54 | 9385 | 9125 | 7.5 | 18 | 7 | G - B | K2 L7 M2 - K1 L7 M2 N1 - K2 L7 M1 N1 | 2 | 37 | 61 |
| 56 | 9140 | 9420 | 15.0 | 19 | 7 | B - G | K2 L7 M0 N1 - K1 L7 M1 N1 - K2 L7 M1 | 3 | | |
| 66 | 9195 | 8142 | 2.5 | 18 | 6 | B - A | K2 L6 M3 - K1 L6 M4 - K2 L5 M4 | 3 | | |
| 67 | 9220 | 8151 | 2.5 | 19 | 6 | B - A | K2 L6 M2 - K1 L6 M3 - K2 L5 M3 | 3 | 1 | 21 |
| 68 | 9515 | 8150 | 1.5 | 19 | 6 | G - A | K2 L6 M2 - K1 L6 M2 N1 - K2 L5 M2 N1 | 2 | 6 | 24 |
| 70 | 9245 | 8154 | 2.5 | 20 | 6 | B - A | K2 L6 M1 - K1 L6 M2 - K2 L5 M2 | 3 | 4 | 29 |
| 71 | 9275 | 8159 | 2.5 | 21 | 6 | B - A | K2 L6 M0 - K1 L6 M1 - K2 L5 M1 | 3 | | |
| 72 | 9635 | 8192 | 1.5 | 21 | 5 | Bh - A | K1 L6 M1 - K0 L6 M2 - K1 L5 M2 - K2 L4 M2 | 2 | | |
| 73 | 9610 | 8434 | 1.5 | 20 | 6 | Bh - Ah | K1 L6 M2 - K0 L6 M3 - K1 L5 M3 | 1 | | |
| 74 | 9460 | 9179 | 12.5 | 16 | 6 | G - B | K2 L6 M5 - K1 L6 M5 N1 - K2 L6 M4 N1 | 1 | | |
| 75 | 9535 | 9229 | 7.5 | 20 | 6 | G - B | K2 L6 M1 - K1 L6 M1 N1 - K2 L6 M0 N1 | 2 | | |
| 76 | 9220 | 9511 | 17.5 | 19 | 6 | B - G | K2 L6 M1 N1 - K1 L6 M2 N1 - K2 L6 M2 | 2 | | |
| 77 | 9245 | 9537 | 15.0 | 20 | 6 | B - G | K2 L6 M0 N1 - K1 L6 M1 N1 - K2 L6 M1 | 1 | | |
| 83 | 9275 | 8185 | 3.5 | 19 | 5 | B - A | K2 L5 M3 - K1 L5 M4 - K2 L4 M4 | 1 | | |
| 87 | 9690 | 8195 | 1.5 | 22 | 5 | Bh - A | K1 L5 M1 - K0 L5 M2 - K1 L5 M1 - K2 L4 M1 | 1 | 7 | 20 |
| 88 | 9345 | 8195 | 1.5 | 21 | 5 | B - A | K2 L5 M1 - K1 L5 M2 - K2 L4 M2 | 2 | | |
| 93 | 9370 | 8198 | 2.5 | 22 | 5 | B - A | K2 L5 M0 - K1 L5 M1 - K2 L4 M1 | 2 | | |
| 95 | 9690 | 8474 | 4.5 | 22 | 5 | Bh - Ah | K1 L5 M1 - K0 L5 M2 - K1 L4 M2 | 1 | | |
| 96 | 9610 | 9295 | 7.5 | 19 | 5 | G - B | K2 L5 M3 - K1 L5 M3 N1 - K2 L5 M2 N1 | 2 | | |
| 97 | 9635 | 9319 | 7.5 | 20 | 5 | G - B | K2 L5 M2 - K1 L5 M2 N1 - K2 L5 M1 N1 | 2 | 31 | 74 |
| 98 | 9360 | 9680 | 20.0 | 21 | 5 | B - G | K2 L5 M0 N1 - K1 L5 M1 N1 - K2 L5 M1 | 1 | | |
| 99 | 9320 | 9620 | 15.0 | 20 | 5 | B - G | K2 L5 M1 N1 - K1 L5 M2 N1 - K2 L5 M2 | 2 | | |
| 103 | 9410 | 8229 | 2.5 | 22 | 4 | B - A | K2 L4 M1 - K1 L4 M2 - K2 L3 M2 | 2 | 8 | 22 |
| 104 | 9460 | 8232 | 2.5 | 23 | 4 | B - A | K2 L4 M0 - K1 L4 M1 - K2 L3 M1 | 2 | 9 | 25 |
| 107 | 9385 | 9720 | 17.5 | 21 | 4 | B - G | K2 L4 M1 N1 - K1 L4 M2 N1 - K2 L4 M2 | 2 | | |
| 108 | 9815 | 8237 | 1.5 | 24 | 4 | Bh - A | K1 L4 M0 - K0 L4 M1 - K1 L4 M0 - K2 L3 M0 | 3 | 12 | 24 |
| 109 | 9765 | 8490 | 5.0 | 23 | 4 | Bh - Ah | K1 L4 M1 - K0 L4 M2 - K1 L3 M2 | 1 | | |
| 110 | 9815 | 8518 | 2.5 | 24 | 4 | Bh - Ah | K1 L4 M0 - K0 L4 M1 - K1 L3 M1 | 3 | | |
| 111 | 9815 | 9423 | 15.0 | 22 | 4 | G - B | K2 L4 M1 - K1 L4 M1 N1 - K2 L4 M0 N1 | 2 | 44 | 107 |
| 115 | 9515 | 8276 | 2.0 | 23 | 3 | B - A | K2 L3 M1 - K1 L3 M2 - K2 L2 M2 | 3 | | |
| 116 | 9535 | 8280 | 1.5 | 24 | 3 | B - A | K2 L3 M0 - K1 L3 M1 - K2 L2 M1 | 3 | | |
| 117 | 9915 | 8278 | 2.0 | 25 | 3 | Bh - A | K1 L3 M0 - K0 L3 M1 - K1 L3 M0 - K2 L2 M0 | 2 | | |
| 118 | 9915 | 8565 | 2.5 | 25 | 3 | Bh - Ah | K1 L3 M0 - K0 L3 M1 - K1 L2 M1 | 2 | | |
| 122 | 9965 | 8567 | 3.5 | 25 | 2 | Bh - Ah | K1 L2 M1 - K0 L2 M2 - K1 L1 M2 | 1 | | |
| 123 | 9690 | 8351 | 2.5 | 26 | 1 | B - A | K2 L1 M0 - K1 L1 M1 - K2 L0 M1 | 2 | | |

TABLE II: Measured resonances and their identification for $I = 110$ kJ/cm$^2$.

## References


[1] T.R. Preston, S. Göde, J.-P. Schwinkendorf, K. Appel, E. Brambrink, V. Cerantola, H. Höppner, M. Makita, A. Pelka, C. Prescher, K. Sukharnikov, A. Schmidt, I. Thorpe, T. Toncian, A. Amouretti, D. Chekrygina, R.W. Falcone, K. Falk, L.B. Fletcher, E. Galtier, M. Harmand, N.J. Hartley, S.P. Hau-Riege, P. Heimann, L.G. Huang, O.S. Humphries, O. Karnbach, D. Kraus, H.J. Lee, B. Nagler, S. Ren, A.K. Schuster, M. Smid, K. Voigt, M. Zhang, and U. Zastrau. Design and performance characterisation of the hapg von hámos spectrometer at

the high energy density instrument of the european xfel. *Journal of Instrumentation*, 15(11):P11033, nov 2020.

[2] X. Pan, M. Šmíd, R. Štefaníková, F. Donat, C. Baehtz, T. Burian, V. Cerantola, L. Gaus, O. S. Humphries, V. Hajkova, L. Juha, M. Krupka, M. Kozlová, Z. Konopkova, T. R. Preston, L. Wollenweber, U. Zastrau, and K. Falk. Imaging x-ray spectrometer at the high energy density instrument of the European x-ray free electron laser. *Review of Scientific Instruments*, 94(3):033501, 03 2023.

[3] J. Chalupský, P. Boháček, T. Burian, V. Hájková, S. P. Hau-Riege, P. A. Heimann, L. Juha, M. Messerschmidt, S. P. Moeller, B. Nagler, M. Rowen, W. F. Schlotter, M. L. Swiggers, J. J. Turner, and J. Krzywinski. Imprinting a focused x-ray laser beam to measure its full spatial characteristics. *Phys. Rev. Appl.*, 4:014004, Jul 2015.

[4] Tobias Dornheim, Maximilian Böhme, Dominik Kraus, Tilo Döppner, Thomas R. Preston, Zhandos A. Moldabekov, and Jan Vorberger. Accurate temperature diagnostics for matter under extreme conditions. *Nature Communications*, 13(1):7911, Dec 2022.

[5] Tobias Dornheim, Maximilian P. Böhme, David A. Chapman, Dominik Kraus, Thomas R. Preston, Zhandos A. Moldabekov, Niclas Schlünzen, Attila Cangi, Tilo Döppner, and Jan Vorberger. Imaginary-time correlation function thermometry: A new, high-accuracy and model-free temperature analysis technique for x-ray Thomson scattering data. *Physics of Plasmas*, 30(4):042707, 04 2023.

[6] M. F. Gu and P. Beiersdorfer. Stark shift and width of x-ray lines from highly charged ions in dense plasmas. *Phys. Rev. A*, 101:032501, Mar 2020.

[7] N. Hell, G. V. Brown, J. Wilms, V. Grinberg, J. Clementson, D. Liedahl, F. S. Porter, R. L. Kelley, C. A. Kilbourne, and P. Beiersdorfer. Laboratory Measurements of the K-shell Transition Energies in L- shell Ions of SI and S. *Astrophys. J.*, 830(1):26, October 2016.

[8] René Steinbrügge, Steffen Kühn, Fabrizio Nicastro, Ming Feng Gu, Moto Togawa, Moritz Hoesch, Jörn Seltmann, Ilya Sergeev, Florian Trinter, Sonja Bernitt, Chintan Shah, Maurice A. Leutenegger, and José R. Crespo López-Urrutia. X-ray photoabsorption of density-sensitive metastable states in ne vii, fe xxii, and fe xxiii. *The Astrophysical Journal*, 941(2):188, dec 2022.

[9] Hai P. Le, Mark Sherlock, and Howard A. Scott. Influence of atomic kinetics on inverse bremsstrahlung heating and nonlocal thermal transport. *Phys. Rev. E*, 100:013202, Jul 2019.

[10] Stefan P. Hau-Riege. Nonequilibrium electron dynamics in materials driven by high-intensity x-ray pulses. *Phys. Rev. E*, 87:053102, May 2013.

[11] Shenyuan Ren, Yuanfeng Shi, Quincy Y. van den Berg, Muhammad F. Kasim, Hyun-Kyung Chung, Elisa V. Fernandez-Tello, Pedro Velarde, Justin S. Wark, and Sam M. Vinko. Non-thermal evolution of dense plasmas driven by intense x-ray fields. *Communications Physics*, 6(1):99, May 2023.

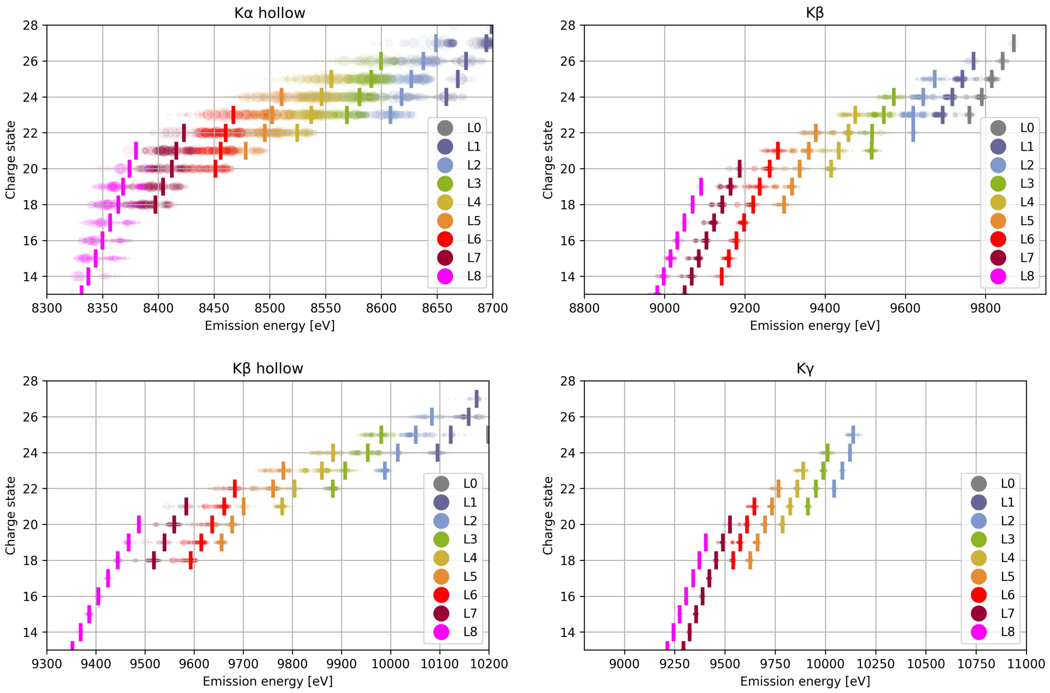


FIG. 8. Line positions calculated by FAC for various lines, see title of respective subfigures.

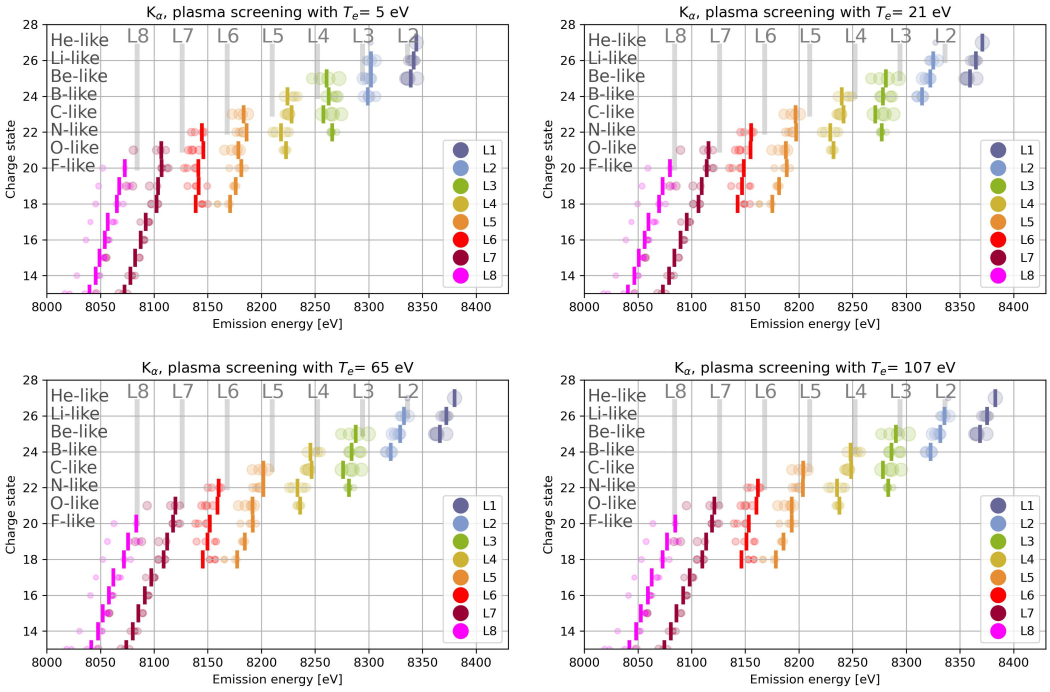


FIG. 9. Energies of Kα calculated by FAC with Stewart Pyatt model of plasma screening with various temperature assumptions, see respective titles. Grey bars are indicating number of electrons in L shell for transitions around that energy.
